# Mid-term results of mitral valve repair for pure mitral regurgitation in children

**DOI:** 10.1186/1749-8090-10-S1-A4

**Published:** 2015-12-16

**Authors:** Abdelmalek Bouzid, Salim Chibane, Mohamed Atbi, Halima Larbi, Boukri Hamouda, Redha Djilali-Sayeh, Youcef Larabi, Tarek Hamdi, Sami Bouchenafa, Ramdan A Ould Abderrahmane

**Affiliations:** 1 Department of Cardiac Surgery, EHU 1er Novembre 54, Oran 31000, Algeria

## Background/Introduction

The surgery of the mitral valve in children is a particular challenge because of the variety of lesions requiring surgical management, feasibility of conservation of the native valve is the first concern of the surgeon

## Aims/Objectives

Our aim is to evaluate the results of conservative mitral valve surgery at mid term.

## Method

10 patients were operated on from January 2009 to January 2014 for lesions of the mitral valve (AVCD were excluded from this study), age was between 04 and 16 years (mean 10 years), 40% male, the lesion was mitral regurgitation in all cases, the lesions were congenital origin in 07 of 10 cases, associated lesions in 20% (2 cases) the presence of atrial septal defect (OS), 10 % (1 case) coarctation of the aorta in 10% (1 case) VSD in 10% (1 cases) and the association of functional tricuspid insufficiency associated requiring treatment.

Various surgical techniques were used according to the functional and pathologic findings of MV.

## Results

There was no hospital or late mortality, 80% (8 cases) of the cases have had good results in the short and medium term (minimal residual insufficiency, good ventricular function, no significant gradient across the mitral valve).

One patient (10%) had a poor outcome, requiring reoperation 34 months after the first surgery with replacement by mechanical prosthesis.

## Discussion/Conclusion

Mitral valve repair in children showed excellent survival, acceptable re-operation rate and satisfactory valve function at mid-term follow-up.

